# The Full-Size ABCG Transporter of *Medicago truncatula* Is Involved in Strigolactone Secretion, Affecting Arbuscular Mycorrhiza

**DOI:** 10.3389/fpls.2020.00018

**Published:** 2020-02-07

**Authors:** Joanna Banasiak, Lorenzo Borghi, Natalia Stec, Enrico Martinoia, Michał Jasiński

**Affiliations:** ^1^Department of Plant Molecular Physiology, Institute of Bioorganic Chemistry, Polish Academy of Sciences, Poznan, Poland; ^2^Department of Plant and Microbial Biology, University of Zurich, Zurich, Switzerland; ^3^Department of Biochemistry and Biotechnology, Poznan University of Life Sciences, Poznan, Poland

**Keywords:** ABC transporters, arbuscular mycorrhiza, exodermis, *Medicago truncatula*, strigolactones, symbioses

## Abstract

Strigolactones (SLs) are plant-derived signaling molecules that stimulate the hyphal branching of arbuscular mycorrhizal fungi (AMF), and consequently promote symbiotic interaction between the fungus and the plant. Currently, our knowledge on the molecular mechanism of SL transport is restricted to the *Solanaceae* family. In the *Solanaceae* family, SL translocation toward the rhizosphere occurs through the exodermis *via* hypodermal passage cells and involves a member of the G subfamily, of the ATP-binding cassette (ABC) membrane transporters. Most *Fabaceae* species, including those that are agriculturally important, have a different root anatomy compared to most angiosperm plants (i.e., lacking an exodermis). Thus, we have investigated how SL transport occurs in the model legume *Medicago truncatula*. Here, we show that overexpression of a SL transporter from petunia (PaPDR1) enhances AMF colonization rates in *M. truncatula*. This result demonstrates the importance of ABCG proteins for the translocation of orobanchol-type molecules to facilitate arbuscular mycorrhiza, regardless of root anatomy and phylogenetic relationships. Moreover, our research has led to the identification of Medicago ABCG59, a close homologue of Petunia PDR1, that exhibits root specific expression and is up-regulated by phosphate starvation as well as in the presence of *rac*-GR24, a synthetic SL. Its promoter is active in cortical cells, root tips, and the meristematic zone of nodules. The *mtabcg59* loss-of-function mutant displayed a reduced level of mycorrhization compared to the WT plants but had no impact on the number of nodules after *Sinorhizobium meliloti* inoculation. The reduced mycorrhization indicates that less SLs are secreted by the mutant plants, which is in line with the observation that *mtabcg59* exudates exhibit a reduced stimulatory effect on the germination of the parasitic plant *Phelipanche ramosa* compared to the corresponding wild type.

## Introduction

Arbuscular mycorrhiza (AM) represents an ancient and widespread beneficial association, established between most terrestrial plants and filamentous fungi from the subphylum *Glomeromycotina* (Parniske, 2008; Spatafora et al., 2016). Arbuscular mycorrhizal fungi (AMF) improve plant nutrient acquisition, especially phosphate, depletion of which is a major factor limiting plant growth (Karandashov and Bucher, 2005). In return, plants supply the fungi with sugars (Doidy et al., 2012) and lipids (Jiang et al., 2017; Luginbuehl et al., 2017; Rich et al., 2017). This nutrient transfer between symbionts occurs *via* highly branched, fungus-derived structures called arbuscules, formed inside root cortical cells (Parniske, 2008; Luginbuehl and Oldroyd, 2017).

The establishment of AM is preceded by a signal exchange between partners that mediates reciprocal recognition, prior to the establishment of direct contact. One of the breakthroughs in the field of AM research was the discovery of strigolactones (SLs) as host-derived precolonization signals (Akiyama et al., 2005). SLs, released into the rhizosphere upon phosphate deficiency, stimulate fungal mitochondrial metabolism, and promote hyphal branching (Akiyama et al., 2005; Besserer et al., 2006). These signaling molecules also contribute to the transcriptional reprogramming of AMF (Tsuzuki et al., 2016) and induce the extrusion of chitin oligosaccharides, the so-called “myc factors”, responsible for the activation of nuclear Ca^2+^ spikes in the host root epidermis (Genre et al., 2013). In the rhizosphere, SLs are active at very low concentrations and are chemically instable. Thus, it has been postulated that SLs form a steep concentration gradient in the soil, indicating the proximity of a plant host and guide fungal hyphae to the root (Akiyama and Hayashi, 2006; Boyer et al., 2012; Nadal and Paszkowski, 2013; Oldroyd, 2013). Moreover, elevated concentrations of SLs near to the root surface may affect the initiation of hyphopodium formation. It has recently been shown that in rice this process is severely attenuated in the SL biosynthetic mutants (Kobae et al., 2018).

Following physical contact and fungal hyphopodium formation on the epidermis, internal root colonization begins. Having penetrated the epidermal cells, the hyphae encounters the hypodermis, the outermost cortical layer. In most angiosperm plants, cell walls within the hypodermis might be modified and form an apoplastic barrier called an exodermis, comprising cells with Casparian bands and suberin lamellae, as well as unsuberized ones. The suberized cells become impenetrable to fungi, therefore deeper cortex colonization occurs only through the suberin-free cells called hypodermal passage cells (HPCs) (Sharda and Koide, 2008). Interestingly, *Petunia hybrida* pleiotropic drug resistance 1 (PhPDR1), the first characterized SL exporter, is specifically expressed in HPCs. Loss-of-function mutations of this gene negatively affects the formation of arbuscular mycorrhiza (Kretzschmar et al., 2012). An orthologue of PhPDR1 from *Petunia axillaris* exhibits a cell-type-specific polar localization in HPCs. PaPDR1 is localized in the outer-lateral plasma membrane of HPCs, in accordance with its proposed function in releasing SL toward the rhizosphere and in creating SL gradients guiding AM fungi to preferred access sites (Sasse et al., 2015). PhPDR1 and PaPDR1 are members of the ancient and ubiquitous ATP-binding cassette (ABC) protein family, and belong to the ABCG subfamily, which is the most abundant in plants and appears to have a great impact on plant adaptations to terrestrial life (Kang et al., 2011; Hwang et al., 2016).

Currently, our knowledge about the molecular mechanisms of SL transport is restricted to the *Solanaceae* family (Kretzschmar et al., 2012; Xie et al., 2015). This is a surprising constraint as SLs influence other types of plant-rhizosphere interactions, both negative and positive. Their presence in the rhizosphere promotes the germination of seeds of parasitic plants of the genera *Striga* and *Orobanche* (Matusova et al., 2005). Recently, a role of SLs in the legume - rhizobium symbiosis (LRS) has also been described. While the function of SL in AM symbiosis is well established, their role in LRS is still emerging. It has been shown, that mutations in the SL biosynthetic genes (*CCD7* and *CDD8*) lead to a decrease in the number of nodules in *Pisum sativum* (pea) and *Lotus japonicus* (Foo and Davies, 2011; Liu et al., 2013). Further analysis in pea revealed that SLs promote the formation of infection threads by influencing the bacterial partner (McAdam et al., 2017). The question about SL transporters seems to be especially intriguing in the case of the leguminous plants (*Fabaceae*), since it was reported that unlike most other angiosperm plants, they do not form an exodermis (Hose et al., 2001; Seago and Fernando, 2013). Moreover, *Fabaceae* such as *Medicago sativa* (alfalfa) and *Glycine max* (soybean) are crops exhibiting an important impact on agriculture and they have evolved several strategies to survive in low nutrient soils (Stagnari et al., 2017). Nitrogen and phosphorus are limiting nutrients in most natural soils. A large part of phosphorus in the soil is not available to plants due to its tendency to interact with cations and it is often present as the sparingly soluble rock phosphate. This non-renewable resource is being mined at an increasing rate to meet the demand for artificial fertilizers (Cordell et al., 2009). In the case of phosphorus, in many ecosystems and, in particular in acidic soils, the response of plants to sparingly available phosphate results mainly in the association with mycorrhizal fungi (Kluber et al., 2012). In this regard it is also worth considering how the root anatomical traits in leguminous plants may influence SL dispersal as well as affect SL coordinated symbiotic processes.

In this study, by functional overexpression of the petunia’s PaPDR1 in *Medicago truncatula*, we have shown that SL transport mechanism, based on ABCG transporters, is conserved between species. Additionally, by analyzing transcriptomic and phylogenetic data, we have identified a full-size ATP-binding cassette (ABC) transporter, namely MtABCG59. This protein is important for establishing mycorrhizal symbiosis in Medicago by acting as an SL exporter localized in the root cortex.

## Materials and Methods

### Plant Materials, Treatments, and Growth Conditions

*Medicago truncatula* seeds were acquired from A. Kondorosi of CNRS, Gif-sur-Yvette, France. Seeds of the *mtabcg59 Tnt1* retrotransposon insertion mutant lines (NF15758 and NF12356) were obtained from the Noble Research Institute. The presence of Tnt1 was confirmed using gene-specific primers and primers annealing to the Tnt1 border (**Supplementary Table 1**).

The Medicago seeds were scarified with 96% sulfuric acid (10 min), stratified (4°C in the dark for 3 days), and were then germinated on solid, half-strength Murashige and Skoog medium (M5524, Sigma-Aldrich). For all experiments plants were grown in a greenhouse (one plant per 0.5 L pot) or growth chamber (*in vitro* experiments) under a 16 h light/8 h dark (24/22°C) regime, at 50%–60% relative humidity.

For the mycorrhization trials the Medicago plants (R-108 and *mtabcg59* mutants) were grown for three weeks (one plant per 0.5 L pot) in clay granules (Oil Dri US-Special, Damolin) with mycorrhizal inoculum (2 g per pot) (Swiss Collection of Arbuscular Mycorrhizal Fungi (SAF)). Clay granules were supplemented once a week with half-strength Hoagland solution. Afterwards the mycorrhizal roots were collected for quantification of AM colonization or AM-marker genes (*MtBCP1* and *MtPT4)* expression analyses. For the experiments five and four biological replicates were performed respectively, 3 independent plants were pooled per replicate.

For the *rac*-GR24 treatments, seven‐day‐old *M. truncatula* seedlings were transferred onto fresh half-strength MS medium supplemented with 10 µM *rac*-GR24 (Chiralix) dissolved in acetone or the equal volume of acetone (mock). Roots were collected 24 h after transfer and immediately frozen. Roots from three plants were pooled for each condition and four independent replicates were performed. The collected material was used for qRT-PCR analyses.

For the phosphorous starvation experiments seven‐day‐old *M. truncatula* seedlings were planted in a mixture of perlite and vermiculite (2:5). Plants were watered twice a week with water and custom prepared fertilizer solution optimized for legumes (**Supplementary Table 2**) or fertilizer without KH_2_PO_4_ and NH_4_H_2_PO_4_. Roots were collected four weeks after they were planted and immediately frozen. Roots from two plants were pooled for each condition and three independent replicates were performed. The collected material was used for qRT-PCR analyses.

### Quantification of Mycorrhization

To determine mycorrhizal colonization, a modified gridline intersect method (Mcgonigle et al., 1990) was applied. Mycorrhizal roots were washed in tap water and boiled for 10 min in 10% KOH. Then the roots were rinsed with ddH_2_O, boiled for 10 min in a solution containing 5% black ink and 5% acetic acid. After washing and de-staining the roots were stored in 5% acetic acid at 4°C. The roots were then spread evenly on a petri dish with 4 mm^2^ grid and the percentage of positive events (roots with intra-radical structures) were calculated from the total amount of segments analyzed. Five independent biological experiments (i.e., 5 pools of 3 plants each) were performed.

### *Phelipanche ramosa* Germination Assay

Rhizotron chambers for assessing the germination of *P. ramosa* seeds were assembled from square petri dishes. Three and one holes were respectively carved on the bottom and on the top of the petri dish to allow for water uptake and plant growth. Ten days after germination on half-strength MS agarose medium, the WT and *mtabcg59* Medicago seedlings were moved onto a round wet filter paper placed in the rhizotron. Then, 150 seeds per filter paper were positioned on the roots and off the roots (seeds placed 1 to 3 mm aside). Another wet filter paper was positioned on the top, creating a paper/roots + seeds/paper sandwich. The rhizotron chambers were then filled up with clay (Oil-Dri), sealed with Micropore Tape (3M) and positioned in a try with water on its bottom to keep the rhizotron moist but not water-logged. Five days later, the rhizotron chambers were opened and germination of *P. ramosa* was quantified. At this stage of development, no significant differences were present for the biomass and surface of seedling roots between the two genotypes (R-108 and *mtabcg59*). No germination was detected without the presence of a plant root. Around 4,000 micro-pilar openings and the eventual presence of radicle were quantified with the help of a binocular microscope.

### Nodulation Assay

For *in vitro* nodulation experiments *M. truncatula* seedlings (R-108, *mtabcg59*) were grown vertically on the surface of the modified solid Fahraeus (-N) medium, pH 6.5. *Sinorhizobium meliloti* strain 1021 was grown overnight at 28°C in liquid Bergensen’s Modified Medium (BMM) containing streptomycin (500 µg/L), and then diluted with sterile BMM to an OD_600_ = 0.1. Six-day-old *M. truncatula* seedling roots were flood-inoculated with 200 µl of bacterial culture spread along the root. Nodule numbers were counted 14 and 21 days after inoculation. There were five replicates (N = 5) with nine plants (n = 9) each, for each genotype.

### Quantitative RT-PCR Analyses

Total RNA of the collected samples were extracted using the RNeasy Plant Mini Kit (Qiagen, Hilden, Germany). The cDNA was then synthesized with an Omniscript Reverse Transcription (RT) Kit (Qiagen) or Moloney Murine Leukemia Virus Reverse Transcriptase Reverse Transcriptase (M-MLV RT) (Promega, Madison, Wisconsin, USA). Quantitative PCR analyses for *MtABCG43*, *MtABCG44*, *MtABCG59, MtCCD7, and MtCCD8* were performed in a CFX Connect Real-Time System machine (BioRad, Hercules, CA, USA) using iTaq Universal SYBR Green supermix (BioRad) with at least three biological replicates each with three technical repeats. Quantitative PCR analyses for the AM-marker genes (*MtBCP1* and *MtPT4*) were performed in a 7500 Fast Real-Time PCR System (Applied Biosystems, Waltham, Massachusetts, USA) using SYBR Green PCR Master Mix (Applied Biosystems) with four biological replicates each with two technical repeats. Expression levels were normalized to *Mtactin* and calculated with the ΔΔCt method. For primer sequences, see **Supplementary Table 1**.

### Genetic Constructs and Plant Transformation

A genomic DNA fragment (1,752 bp) corresponding to the promoter region of *MtABCG59* was amplified with KOD Hot Start DNA Polymerase (Novagen, Madison, Wisconsin, USA), and cloned into the following vectors: (i) pPR97, carrying the β-glucuronidase (gusA) reporter gene (Szabados et al., 1995), by restriction-ligation using the restriction sites for *EcoR*I and *Hind*III; and (ii) pPLV04_v2, carrying a GFP reporter gene tagged with a nuclear localization signal (NLS), by ligation-independent cloning (De Rybel et al., 2011). The genomic DNA fragment (7,500 bp) of *MtABCG59* was used for subcellular localization and was amplified with KOD Hot Start DNA Polymerase (Novagen) and cloned into the Gateway entry vectors pENTR4 (Invitrogen, Carlsbed, CA, USA) by restriction-ligation, using restriction sites for *Not*I and *Sal*I. The resulting entry clone was used for recombination with a destination vector pMDC43 (Curtis and Grossniklaus, 2003) using Gateway LR Clonase II Enzyme Mix (Invitrogen). The gPaPDR1 cloning procedure was described in (Kretzschmar et al., 2012). For primers, see **Supplementary Table 1**.

### Plant Transformation

Transgenic roots carrying *ProMtABCG59:GUS* or *ProMtABCG59:NLS-GFP* constructs were obtained from *M. truncatula* after inoculation of a severed radicle with *Agrobacterium rhizogenes* Arqua1 (at least 30 composite plants for each construct). Stably transformed *M. truncatula* plants carrying *ProMtABCG59:GUS* (6 independent F0 lines) or *Pro35S:GFP-PaPDR1* (3 independent F0 lines) were obtained by *Agrobacterium tumefaciens* AGL1-mediated transformation using leaf explants and regeneration *via* somatic embryogenesis. The *M. truncatula* transformation was performed according to the protocol described in the Medicago handbook (http://www.noble.org/medicagohandbook). Arabidopsis protoplasts that transiently expressed *Pro35S:GFP-MtABCG59* or free GFP were obtained using PEG-mediated direct gene transfer, as described previously (Smolarkiewicz et al., 2014).

### Microscopic Observation and Staining Procedures

Transgenic hairy roots and stably transformed *M. truncatula* (plants of the F1 generation) carrying GUS reporter constructs were stained using 5-bromo-4-chloro-3-indolyl-β-D-glucuronide, as previously described (Gallagher, 1992) and visualized by light microscopy.

Microscopic observations of the transgenic roots carrying the *ProMtABCG59:NLS-GFP* reporter construct and Arabidopsis protoplasts transiently expressing *Pro35S:GFP-MtABCG59* or free GFP were performed using laser scanning confocal microscopy (Leica TCS SP5).

For suberin staining, 6-week-old *M. truncatula* and *Nicotiana tabacum* roots (10 of each species) were incubated in 0.01% solution of Fluorol Yellow 088 (Santa Cruz Biotechnology, Dallas, Texas, USA) at 70°C for 30 min. Free-hand root sections (from the regions located at least 3 cm above the root tip) were observed under UV-light with fluorescence microscopy (Leica DMI 4000B, Wetzlar, Germany).

For arbuscules visualization, mycorrhizal roots were fixed in 50% ethanol for 3 h, cleared in 20% (w/v) KOH for 3 days at room temperature (21°C–23°C), followed by 0.1 M HCl for 1 h. Then roots were washed in PBS and incubated in WGA-AlexaFluor 488 staining solution (1 µg/ml in PBS and 0.01% v/v Tween 20) in the dark for 16 h. The roots were imaged with laser scanning confocal microscopy (Leica TCS SP5).

## Results

### *Medicago truncatula* Root Anatomy

**Figure 1:**
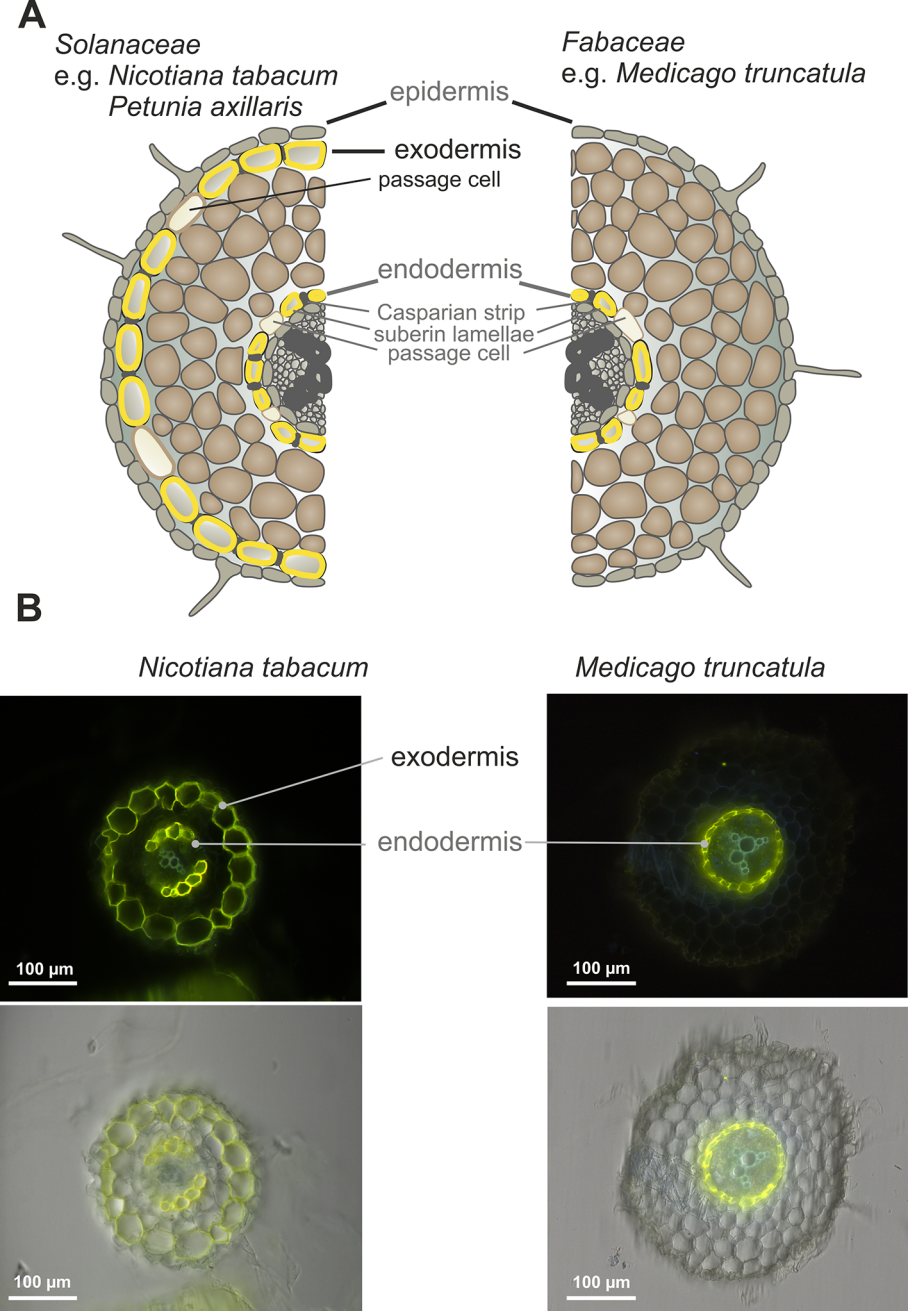
Root anatomical structure. **(A)** Scheme of *Nicotiana tabacum* (*Solanaceae*) and *Medicago truncatula* (*Fabaceae*) root layers. **(B)** Deposition of suberin lamellae in roots of 6-week-old *N. tabacum* and *M. truncatula* (representative images from 10 roots). The cross-sections of the roots comes from the regions located 3 cm above the root tip. Fluoral yellow 088 fluorescence (upper panel), fluorescence images overlayed with corresponding bright-field images (lower panel).

It has been shown that the secretion of SLs in petunia, belonging to the *Solanaceae* family, occurs through the hypodermal passage cells (HPCs), located within the exodermis (Kretzschmar et al., 2012). Notably, most *Fabaceae* species have a different root anatomy and do not form an exodermis (Perumalla et al., 1990; Seago and Fernando, 2013). This is also true for the model legume *Medicago truncatula*, as shown by the Fluorol Yellow 088 fluorescent staining of the suberin lamella in cross-sections of its roots. In contrast to a plant such as *Nicotiana tabacum*, whose roots possess two layers, an exo- and endodermis, which are stained by this dye, Medicago possesses only one layer corresponding to the endodermis (**Figure 1**). The lack of exodermis in Medicago roots has been observed regardless of the plant age or the region of the root examined (**Supplementary Figure 1**).

In petunia, SLs have been shown to be translocated and secreted into the rhizosphere by the membrane transporter PDR1, which belongs to the full-size ABCG (PDR) subfamily of ABC transporters (Kretzschmar et al., 2012). Interestingly, overexpression of the PaPDR1 (the only up to date cloned SL transporter), in Medicago enhanced AMF colonization rates, and did not affect the arbuscule structures in Medicago (**Supplementary Figure 2**). This observation underlines the importance of ABCG proteins for the translocation of orobanchol-type molecules to facilitate AM symbiotic interactions, despite the differences in the anatomical features of the roots between the plant families. The *M. truncatula* transgenic plants overexpressing *PaPDR1* also displayed several SL-related phenotypes in the shoot, demonstrating the importance of SL redistribution in Medicago aboveground organs by this transporter. PaPDR1 OE plants possessed: (i) modified leaf shape with deeper margin serrations, (ii) enhanced internode elongation, and iii) petioles of a reduced length (**Supplementary Figure 3**).

### Identification of a Potential Strigolactone Transporter in *Medicago truncatula*

**Figure 2:**
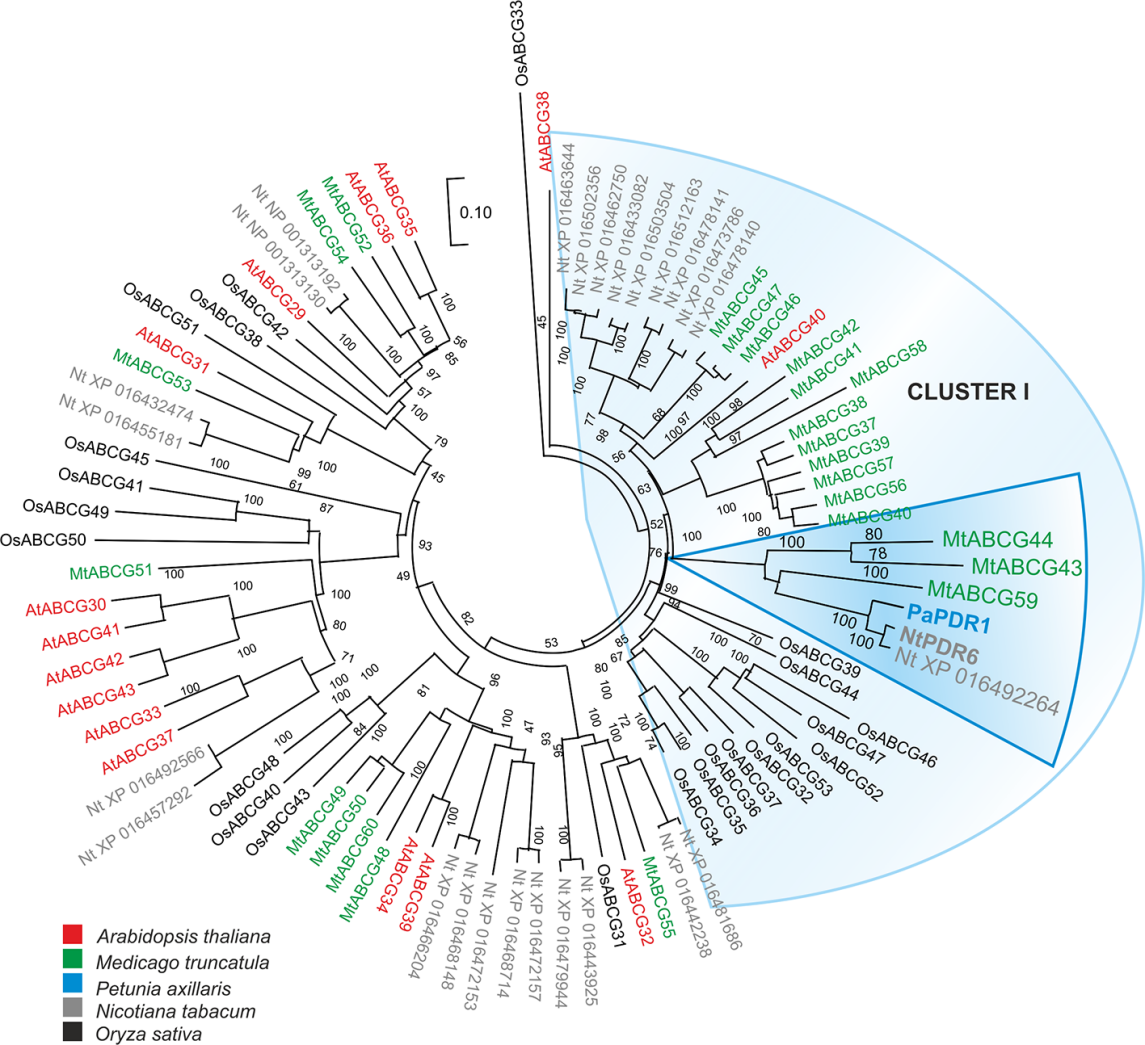
Phylogenetic analysis of full-size ABCG (Pleiotropic drug resistance - PDR) proteins in various plants. Neighbor-joining phylogenetic tree (bootstraps: 1000) was conducted using MEGA X software (Kumar et al., 2018) based on the amino acid sequences generated after multiple sequence alignments with MUSCLE. The highlighted PaPDR1 and NtPDR6, as well as their homologues from *Medicago truncatula* belong to Cluster I (Crouzet et al., 2013).

**Figure 3:**
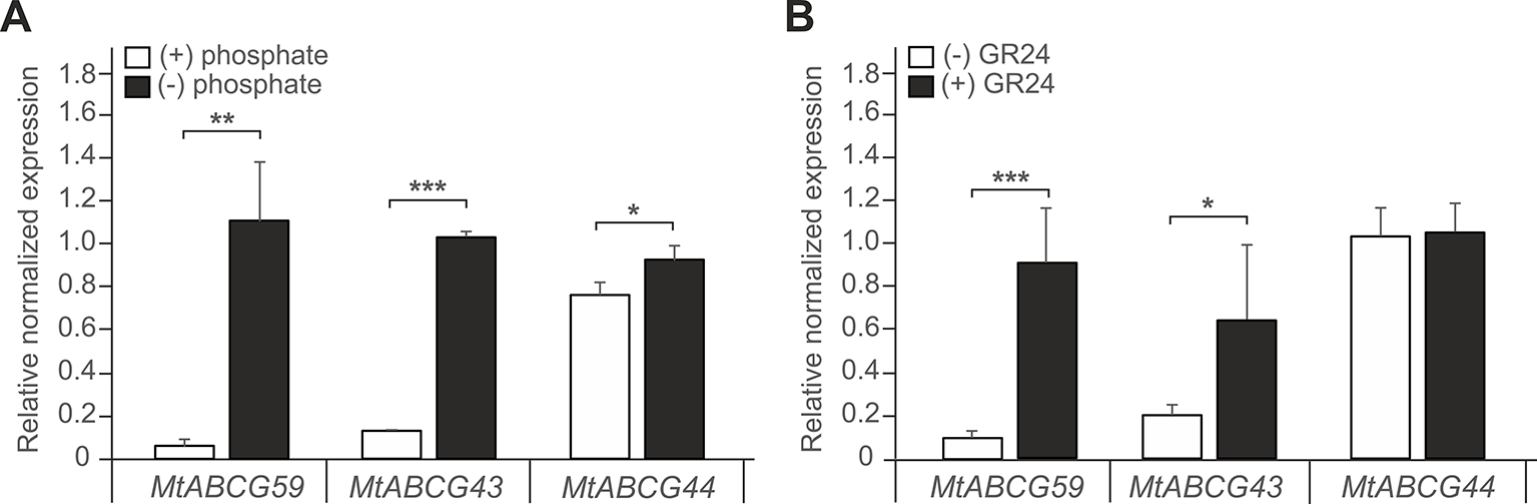
Expression profiles of *Medicago truncatula ABCG59*, *ABCG43*, and *ABCG44*. **(A, B)** Quantitative Real-Time PCR expression analysis of *MtABCG59*, *MtABCG43*, and *MtABCG44* in roots under phosphate-limiting conditions **(A)** or treated with the synthetic strigolactone analogue *rac*-GR24 **(B)**. The transcript levels were normalized to the *actin* gene from *Medicago truncatula*. Data represent the mean ± SD of three and four independent biological experiments, respectively, and three technical repeats. Significant differences from the control plants determined by Student's t-test are indicated as follows: *P < 0.05, **P < 0.01, ***P < 0.001.

We have previously identified several ABCG transporters in the *M. truncatula* genome (Banasiak and Jasinski, 2014). The phylogenetic analysis showed that three ABCG proteins: MtABCG59 (MtPDR23), MtABCG44 (MtPDR8), and MtABCG43 (MtPDR7), clustered with the previously characterized SL transporter PaPDR1 from *P. axillaris* (**Figure 2**, **Supplementary Table 3**). They share 74%, 71%, and 68% amino acid sequence identity with PaPDR1, respectively. Since nutrient starvation stimulates AM formation as well as biosynthesis and secretion of SLs in Petunia (Liu et al., 2018b), we have tested the response of those three genes to phosphorous deficiency. The highest level of gene induction was observed for *MtABCG59* and *MtABCG43* exhibiting 18 and 17-fold changes, respectively (**Figure 3A**). Furthermore, exogenous applications of a synthetic SLs analogue – *rac*-GR24 onto the roots, increased the mRNA accumulation of *MtABCG59* (9-fold) and again at a lower extent *MtABCG43* (3-fold change) (**Figure 3B**). Previously it was reported that the expression of several ABCG transporters could be substrate inducible (Jasinski et al., 2001; Biala et al., 2017; Pawela et al., 2019). The *MtABCG44* was only slightly induced (1.2-fold change) under phosphate limiting condition, and *rac*-GR24 had no impact on its expression (**Figure 3**). Based on this expression profile, and phylogenetic relationships, we selected MtABCG59 for further and more comprehensive functional analyses.

### Gene Expression Pattern of *MtABCG59* in *M. truncatula* and Subcellular Localization of the Corresponding Protein

**Figure 4:**
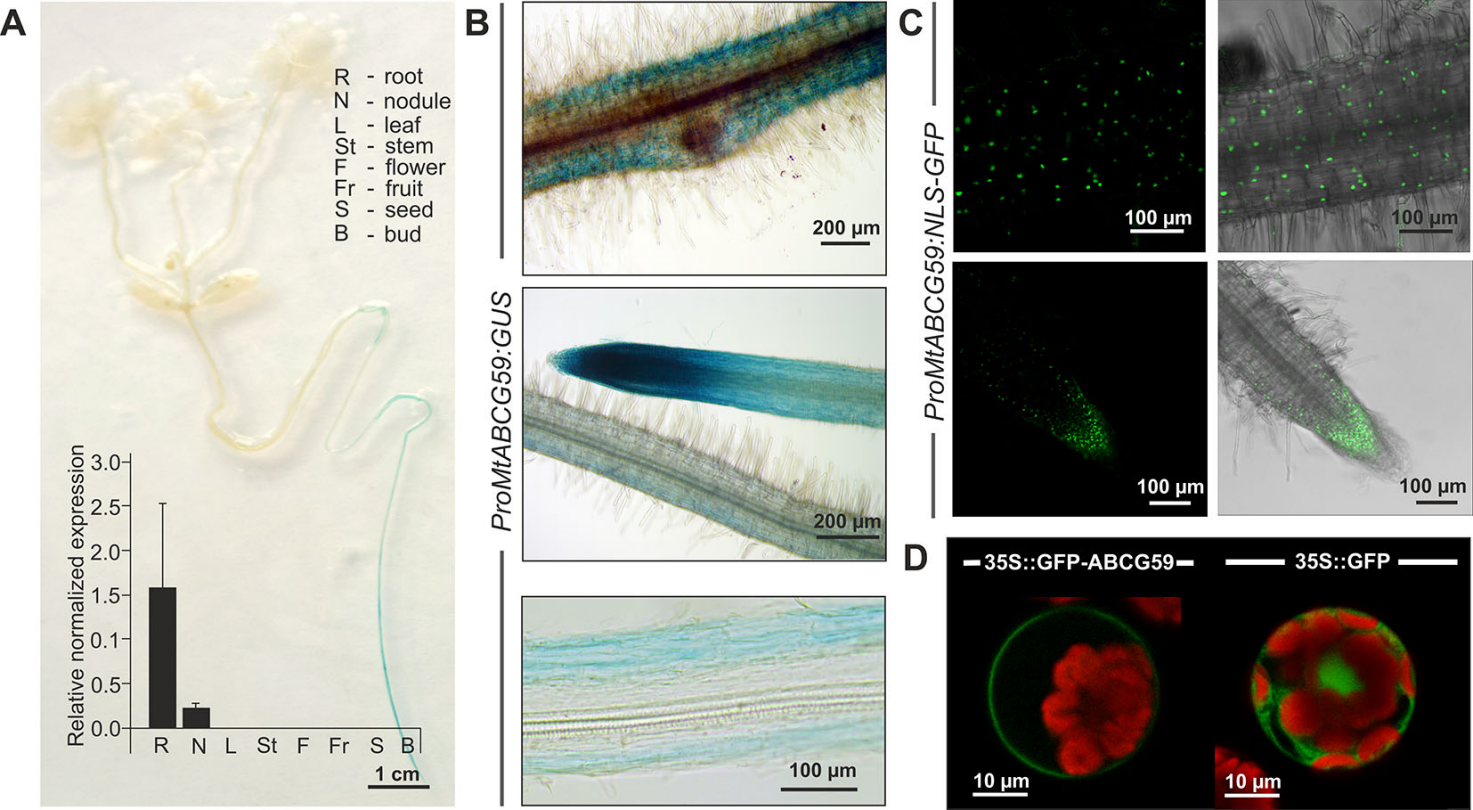
Expression pattern and subcellular localization of MtABCG59. **(A)** GUS staining of 2-week-old transgenic *Medicago truncatula* expressing *ProMtABCG59:GUS* and quantitative PCR expression analysis of *MtABCG59* in different *M. truncatula* organs (three-month-old plants) revealed the *MtABCG59* transcript accumulation exclusively in the roots and nodules. The transcript levels were normalized to the *actin* gene from *M. truncatula*. Data represent the mean ± SD of three independent biological experiments and three technical repeats. **(B)** X-Gluc staining of *ProMtABCG59:GUS* reporter line in root cortex (upper panel) and in root meristem (middle panel), X-Gluc staining of root cross-sections (bottom panel). **(C)** Fluorescence signal of *ProMtABCG59:NLS-GFP* reporter line in root cortex (upper panel) and in root meristem (bottom panel). **(D)** Arabidopsis mesophyll protoplast expressing the fusion gene *Pro35S:GFP-MtABCG59*. The GFP signal was observed in the plasma membrane (left panel). Control Arabidopsis protoplast expressing free cytoplasmic GFP (right panel). The red color represents chlorophyll autofluorescence.

The qPCR analyses revealed that *MtABCG59* mRNA accumulates in roots and root-derived nodules (**Figure 4A**). To further explore the spatial expression patterns of *MtABCG59*, we generated *M. truncatula* transgenic plants expressing the β-glucuronidase (GUS) reporter gene under the control of the native *MtABCG59* promoter (*ProMtABCG59:GUS*). Histochemical staining of the 2-week-old transgenic seedlings, as well as qPCR, showed that *MtABCG59* expression is root specific (**Figure 4A**). Moreover, microscopic observations of transgenic Medicago hairy roots expressing *ProMtABCG59:GUS* and *ProMtABCG59* fused with a nuclear-localized version of green fluorescent protein (*ProMtABCG59:NLS-GFP*) revealed that the root tip, where SLs are biosynthesized (van Zeijl et al., 2015a), and the cortical cells, are the main sites of *MtABCG59* promoter activity (**Figures 4B, C**).

To determine the subcellular localization of MtABCG59, we fused GFP to the C-terminal end of MtABCG59 and transiently expressed the *Pro35S:GFP-MtABCG59* recombinant gene in Arabidopsis leaf mesophyll protoplasts. Visualization of GFP fluorescence was performed using confocal microscopy. The GFP signal from the fusion protein was detected in the plasma membrane. In control protoplasts, transformed with *Pro35S:GFP*, the fluorescence signal was observed in the cytoplasm (**Figure 4D**).

### Mycorrhizal Phenotype of *mtabcg59* Mutant Lines

**Figure 5:**
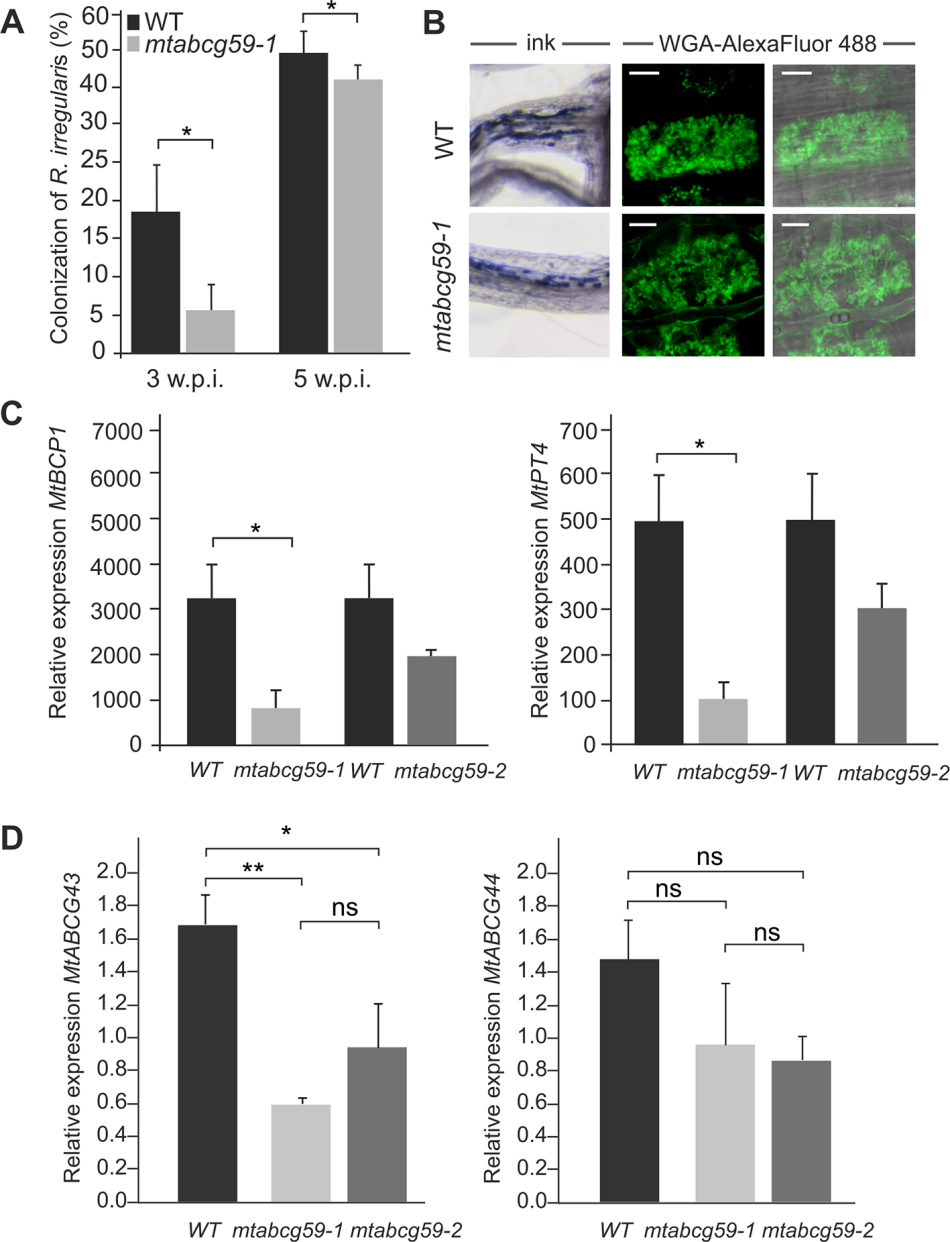
Phenotypic characterization of the *mtabcg59* mutant in mycorrhization contexts. **(A)** Mycorrhizal colonization of *Medicago truncatula* control (WT) and *mtabcg59-1* mutant roots 3 and 5 weeks after inoculation with *Rhizophagus irregularis*. The percentage of the root length colonized by the mycorrhizal fungi is shown. Data represent the means ± SE of five independent biological experiments (i.e. 5 pools of 3 plants each). Significant differences from the control plants were determined by Student's t-test and are indicated by: *P < 0.05. **(B)** Arbuscules formed in the WT and *mtabcg59-1* mutant were morphologically similar. Ink-staining of fungal structures (left panel), WGA-AlexaFluor 488 staining of arbuscules (right panel), scale bar, 10 µm. **(C)** Transcript accumulation of AM-related marker genes *MtBCP1* (left panel) and *MtPT4* (right panel) in *mtabcg59* mutants and control (WT) roots measured by quantitative real-time PCR. The data represents the mean ± SD of four independent biological experiments and two technical repeats. Significant differences from the control plants were determined by Student's t-test and are indicated by: * P < 0.05. **(D)** Relative expression of *MtABCG43* and *MtABCG44* in WT and *mtabcg59* mutant plants. Values represent the mean of three biological replicates ± SD. Significant differences in genes expression between WT and *mtabcg59* mutants were determined by an ANOVA test and Tukey's multiple comparison test, and are as follows: ns, not significant; *P < 0.05, **P < 0.01.

To test the hypothesis that MtABCG59 can influence the AM symbiosis we identified the tobacco retrotransposon (Tnt1) inserted line (NF15758) for *MtABCG59* and assessed with the grid-line intersect method (see M&M) the colonization rate of AMF in the roots derived from the WT and *mtabcg59-1*. The percent of root length colonization by *Rhizophagus irregularis* was estimated 3 and 5 weeks after inoculation with the fungal spores. The experiments showed that the *mtabcg59-1* mutant exhibits 70% and 11% lower levels of colonization than the WT, respectively (**Figure 5A**). The arbuscule structures appeared unaffected in *mtabcg59-1*, suggesting that MtABCG59 is required for initiation of AM, rather than proper development of arbuscules (**Figure 5B**). The reduction of AM formation in *mtabcg59-1* as well as in the second mutant line (*mtabcg59-2*, NF12356) 3wpi was further demonstrated with the usage of AM-related marker genes *MtBCP1* and *MtPT4* (**Figure 5C**). *MtBCP1*, encoding a blue copper protein, is expressed in the epidermal and cortical cells containing fungal structures and in adjacent noncolonized cortical cells (Hohnjec et al., 2005; Hogekamp and Kuster, 2013; Zhang et al., 2015), while *MtPT4* encodes an arbuscule-specific phosphate transporter (Javot et al., 2007; Hogekamp and Kuster, 2013). Real-time PCR analyses revealed that AM marker genes had lower expression levels in the *mtabcg59-1* and *mtabcg59-2*, reflecting the lower mycorrhization rate in these plants compared to the WT (**Figure 5C**).

Taking into the consideration the possible functional redundancy, we have investigated if MtABCG59 paralogues, namely MtABCG43 and MtABCG44 might take over the function of MtACG59. Interestingly, we have observed that upon phosphate deficient condition *MtABCG43* is downregulated in both *mtabcg59* mutants background. Concomitantly, the expression of *MtABCG44* did not show significant differences (**Figure 5D**).

### Root Exudates From *mtabcg59* Exhibit a Reduced Ability to Promote Germination of *Phelipanche ramosa* Seeds

**Figure 6:**
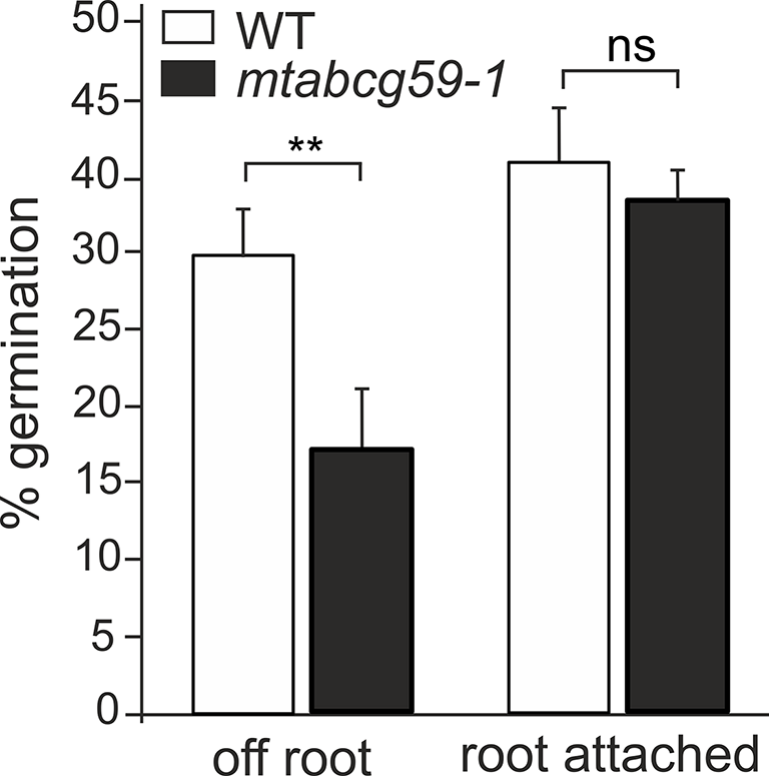
Germination rates of *Phelipanche ramosa* seeds exposed to WT and *mtabcg59-1* exudates. Root attached (seeds placed directly in root surface); off root (seeds placed 1 to 3 mm aside). The data represent the mean ± SE of six to seven independent biological experiments (approx. 150 seeds screened for each biological replicate). Significant differences from the control plants were determined by Student's t-test and are indicated by: ns, not significant; **P < 0.005.

To investigate whether MtABCG59 is involved in SL secretion, *mtabcg59-1* root exudates were assessed for their ability to stimulate parasitic weed *Phelipanche ramosa* seed germination. Compared to the WT, a significant decrease in germination capacity was observed for *P. ramosa* seeds exposed to root exudates from *mtabcg59-1*. Interestingly, this difference was present only with seeds placed 1 to 3 mm aside, no difference in germination rates were observed with seeds placed directly on the root surface (**Figure 6**, **Supplementary Figure 4**).

### Lack of MtABCG59 Disturbs Strigolactone Biosynthesis

**Figure 7:**
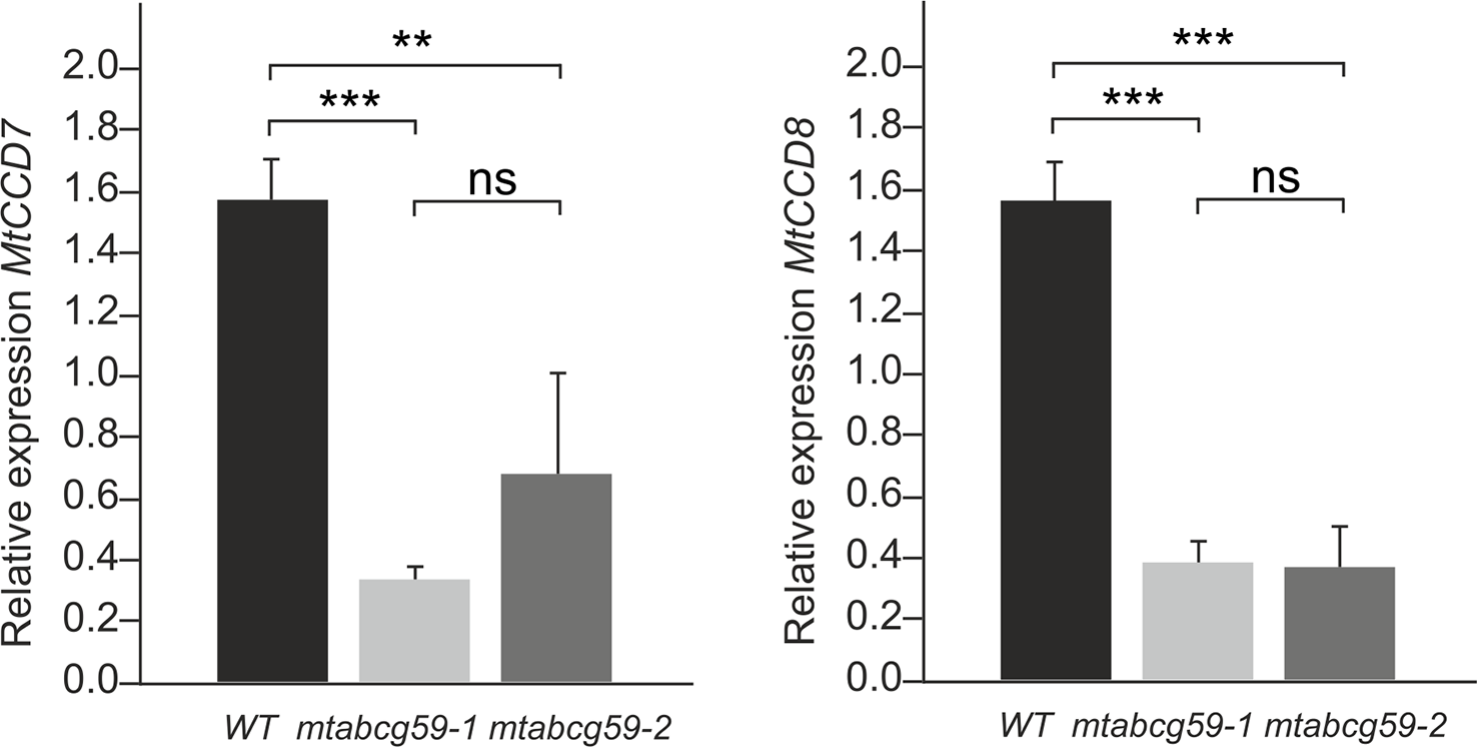
MtABCG59 affects the SL-biosynthetic pathway. Relative expression of SL-biosynthesis gene (*MtCCD7* and *MtCCD8*) in WT and *mtabcg59* mutant plants. Values represent the mean of three biological replicates ± SD. Significant differences in genes expression between WT and *mtabcg59* mutants were determined by an ANOVA test and Tukey's multiple comparison test, and are as follows: ns, not significant; **P < 0.01, ***P < 0.001.

The presence of *MtABCG59* transcripts in root tips suggests that MtABCG59 might be involved in the export of SL from the site of biosynthesis. Since SL content can influence the transcript level of SL biosynthetic genes, *CCD7* and *CCD8* (Foo et al., 2005; Hayward et al., 2009) we have examined their expression in WT and *mtabcg59* mutant lines. We have noticed a decrease in mRNA accumulation of *CCD7* and *CCD8* in both *mtabcg59* mutants background. The observed effect might be due to excessive accumulation of SL in the cells that can trigger a negative feedback regulation mechanism in SL biosynthesis (**Figure 7**).

### Lack of MtABCG59 Does Not Affect Nodulation

**Figure 8:**
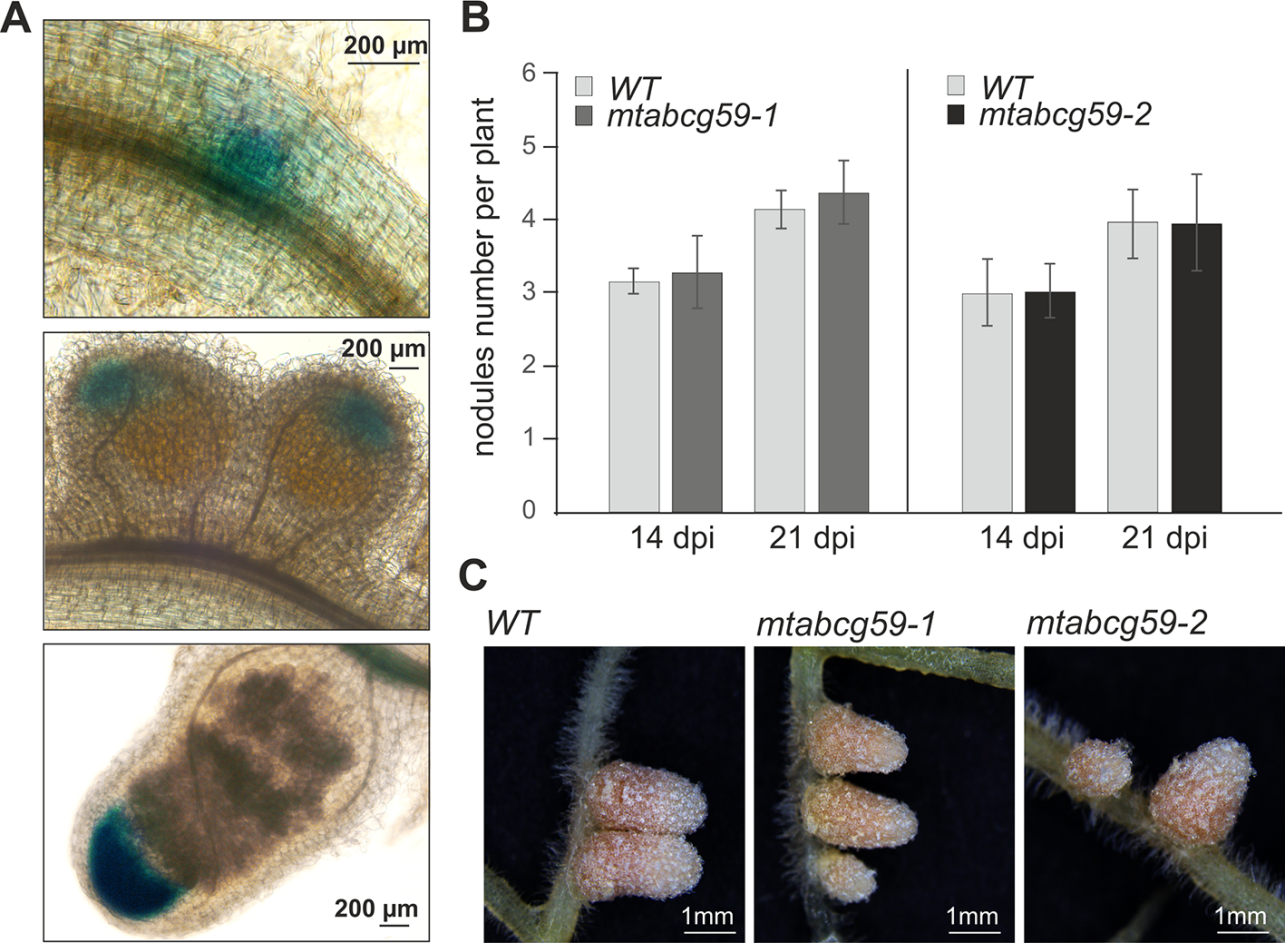
Phenotypic characterization of *mtabcg59* in nodulation context. **(A)** Expression of *ProMtABCG59:GUS* in nodules. **(B)** Average nodule number per plant in WT and *mtabcg59* plants. Six-day-old seedlings were inoculated with *Sinorhizobium meliloti* and grown on modified Fahraeus (-N) medium. At 14- and 21-days post-inoculation (dpi), the nodules were quantified. The data represent the mean ± SD of (N = 5, n = 9), per line. **(C)** WT and *mtabcg59* nodules morphology.

Recent studies have found that SLs positively affect interactions between legumes and nitrogen-fixing bacteria (McAdam et al., 2017). Moreover, we have shown that the expression pattern of *MtABCG59* in nodule primordia as well as in the meristematic zone of developing and mature nodules (**Figure 8A**) is similar to that reported for genes encoding SL biosynthetic enzymes (van Zeijl et al., 2015a). Despite this, *mtabcg59* plants inoculated with *Sinorhizobium meliloti* had a comparable number of root nodules to the WT, and did not differ morphologically (**Figures 8B, C**), indicating that MtABCG59 is either not required for nodulation or that a redundant, yet unknown protein compensates its loss of function.

## Discussion

The only SL transporter characterized in detail is the PaPDR1 from *Petunia axillaris*, and it operates in a highly cell specific manner, being active *inter alia* in the hypodermal passage cells (HPCs) located within exoderemis (Sasse et al., 2015). We have shown that the roots of the model legume plant *Medicago truncatula*, related to the important forage crop *Medicago sativa*, do not possess an exodermis and HPCs (**Figure 1**). Overexpression of *PaPDR1* in Medicago revealed that despite these anatomical differences, this transporter is functional in Medicago and its presence promotes AM. The increased mycorrhization in Medicago overexpressing *PaPDR1*, indicates that SL transport mechanisms based on ABCG transporters, exhibit similar properties between species. Notably, canonical orobanchol-type molecules are the most abundant SLs exuded into the rhizosphere by both petunia and *M. truncatula* (Kretzschmar et al., 2012; Tokunaga et al., 2015). Thus, the PaPDR1 OE strategy appears to be useful for ameliorating mycorrhization rate in *Fabaceae*. It could improve phosphate acquisition and plant biomass production as it was reported for petunia (Liu et al., 2018a; Liu et al., 2018b).

It has been demonstrated that AMF hyphal branching can be initiated by the SL present in the root exudates of legume plants (Akiyama et al., 2005; Steinkellner et al., 2007). However, transporters responsible for SL release into the rhizosphere from this plant family have not been discovered. To identify a putative SL transporter in *M. truncatula*, we combined the phylogenetic information with expression profile analyses. Among the tested genes, *MtABCG59*, besides sharing the highest level of sequence identity with the previously described SL exporter PaPDR1 from petunia (**Figure 2**), it had the highest induction rates in roots in phosphate limited conditions and after *rac*-GR24 treatment (**Figure 3**). We have shown that in Medicago roots, the expression of the putative SL transporter MtABCG59 occurs mainly in the outer cortical cells (**Figure 4B**). The observed expression pattern is compatible with the proposed function of MtABCG59 in SL secretion from the roots toward the soil to induce hyphal branching of AM fungi and guide them to host roots. As *MtABCG59* is also expressed in Medicago colonized roots (**Supplementary Figure 5**), it cannot be excluded that it also participates in the SL translocation to the apoplast, guiding intraradical hyphal branching within the root cortex. Additionally, the presence of *MtABCG59* transcripts in root tips suggests that MtABCG59 might be involved in the export of SL from the site of biosynthesis towards the upper root, as has been previously revealed for PaPDR1 (Sasse et al., 2015). This hypothesis can be supported by the observation that SL-biosynthesis genes *MtCCD7* and *MtCCD8* are downregulated in *mtabcg59* mutant lines compared to WT (**Figure 7**, **Supplementary Figure 6**). Previously, it was proposed that excessive accumulation of SL in the cells can trigger a negative feedback regulation mechanism in SL biosynthesis and affect mRNA accumulation of *CCD7* and *CCD8* (Foo et al., 2005; Hayward et al., 2009; Liu et al., 2018b).

**Figure 9:**
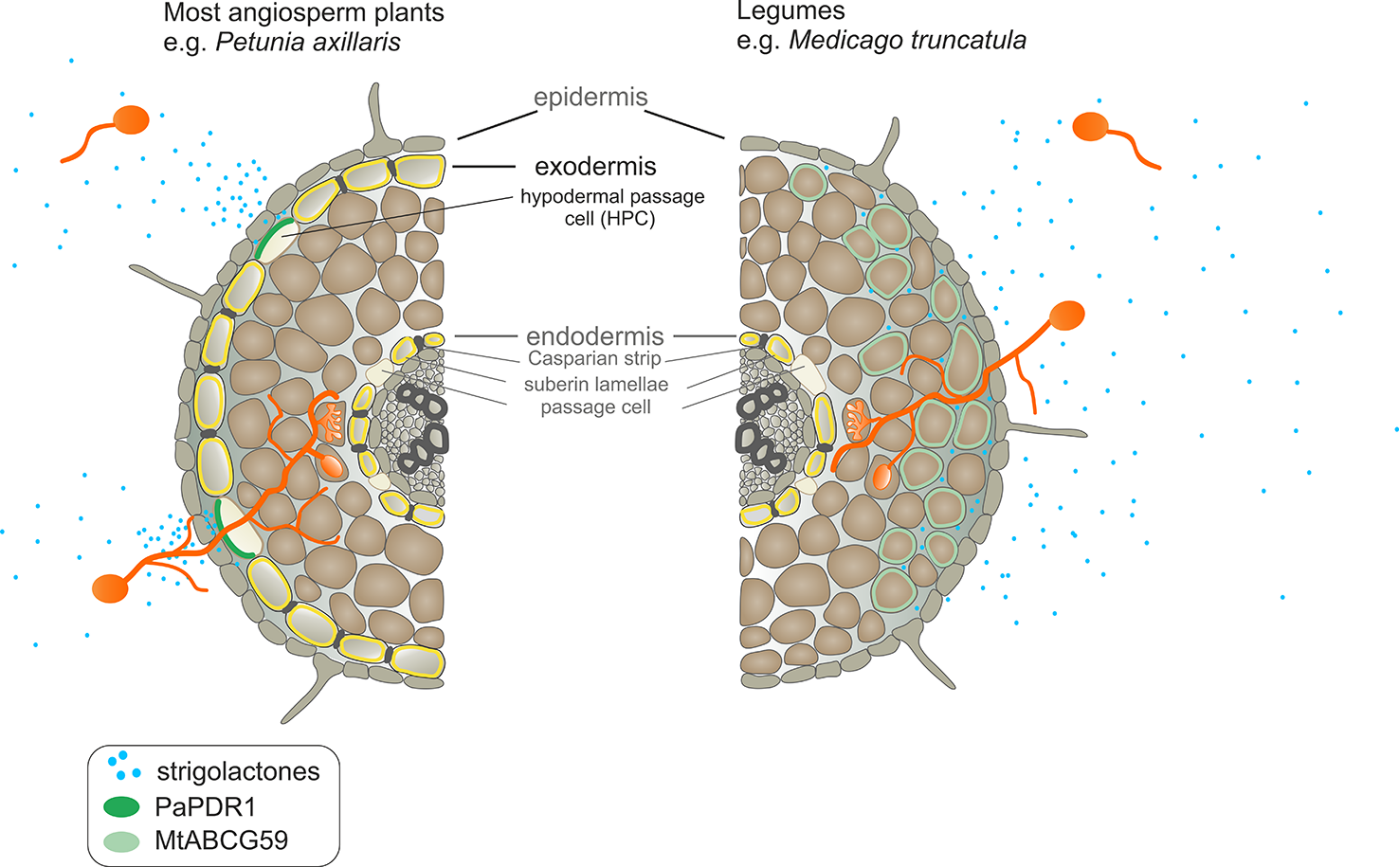
Comparison of strigolactone (SL) exudation into the soil and SL-dependent guidance of arbuscular mycorrhizal fungi to the host root between *Petunia axillaris* and *Medicago truncatula*. In *P. axillaris*, SLs are secreted into the rhizosphere by PaPDR1 that is localized in the hypodermal passage cells (HPCs) within the exodermis. A steep concentration gradient of SLs, created by PaPDR1, guides arbuscular mycorrhizal fungi (AMF) to access penetration sites – i.e. unsuberized HPCs. In the *M. truncatula*, due to a lack of exodermis, SLs could passively enter the rhizosphere because they do not encounter the apoplastic diffusion barrier. However, we suggest that to achieve the full extent of AMF colonization, the active export of SLs, mediated by MtABCG59, is required. Its action ensures sufficiently high concentrations of SLs around the roots to attract AM fungi, which together with the characteristic root anatomical traits in *M. truncatula*, enables the colonization of the whole root surface.

Phenotypic characterization of the *mtabcg59-1* mutant showed that its loss of function negatively influences AM formation, especially, at the early stage of interaction. Previously, it was proposed that altered presymbiotic AM fungal growth could result in delayed colonization, which is apparent at early time points (Montero et al., 2019). That is also what we observed for *mtabcg59* mutant (**Figure 5**). Likewise, as in other reported SL-related mutants (e.g., biosynthetic, perception, and transport) (Kretzschmar et al., 2012; Lanfranco et al., 2018), the arbuscular structure in colonized root cells was not altered in *mtabcg59* plants (**Figure 5**). This observation supports the notion that SLs influence the fungal morphology mainly in the rhizosphere but do not affect its intercellular accommodation and development. SLs *ex planta* stimulate the seed germination of parasitic weeds such as witchweeds (Striga spp.) and broomrapes (*Orobanche* and *Phelipanche* spp.) (Cook et al., 1966; Yoneyama et al., 2018). To verify whether the decrease of AM formation in *mtabcg59-1* is indeed caused by a reduction in the secretion of SLs, we applied a parasitic seed germination bioassay. This approach allows for the indirect but sensitive determination of the relative amounts of SLs exuded by roots (Guillotin et al., 2017). The SL levels secreted by the *mtabcg59-1* were assessed by the germination of *Phelipanche ramosa* seeds that exhibit a broad host range, including *M. truncatula* (Fernandez-Aparicio et al., 2009). Our experiments revealed that root exudates derived from *mtabcg59-1* possess lower germination-inducing activity than exudates from the WT. Interestingly, the effect observed concerned only seeds which were not directly in touch with the roots. Further studies are needed to test whether this lack of difference in seed germination in seeds that are in touch with the root is due to a second, less active transporter or due to diffusion of the hydrophobic SLs. In Medicago apart from *MtABCG59*, expression of *MtABCG43* is induced by phosphorous deficiency and *rac*-GR24 (**Figure 3**). Further investigation is required to demonstrate that MtABCG43 participates in SL secretion, however, functional redundancy is possible. Nevertheless, since the expression of *MtABCG43* decreased in *mtabcg59* mutants compared to WT (**Figure 5D**), the diffusion of SL through the exodermis-free cortex appears to be more likely. In the case of *mtabcg59* mutant, the concentration of diffusion-derived SL on the root surface is still sufficient for parasitic seed germination. Taken together, our findings suggest that MtABCG59 is involved in SL extrusion to the soil to maintain an SL concentration sufficiently high in the proximity of the roots, allowing to induce hyphal branching and guiding the fungi towards the host root (**Figure 9**).

In addition to arbuscular mycorrhiza, SLs are implicated in the legume-rhizobium symbiosis (LRS) (Lopez-Raez et al., 2017). A characteristic feature of this type of interaction is the formation of root nodules, which are the place of nitrogen fixation and nutrient exchange between partners. Phenotypic analyses of SL biosynthetic mutants of *Pisum sativum*, *Lotus japonicus*, and *Glycine max* demonstrated the positive role of SLs in LRS establishment (Foo and Davies, 2011; Liu et al., 2013; Rehman et al., 2018). In the case of *M. truncatula*, exogenous application of *rac*-GR24 had either a positive or negative effect on this process, depending on the SL concentrations used (De Cuyper et al., 2015). We could not detect a significant difference in the root nodule structures or the number of root nodules between the *mtabcg59* and WT despite the fact that *MtABCG59* is expressed in the nodule meristem (**Figure 8**) and co-expressed with the SL biosynthetic genes (*MtD27*, *MtCCD7*, and *MtCDD8*) (van Zeijl et al., 2015a). Although SLs are biosynthesized in mature nodules, their role in this organ remains currently unknown. Pea SL-deficient mutants produced less nodules, but their size and ability to fix atmospheric nitrogen were comparable to those of WT plants. Therefore, it was postulated that SLs affect the LRS mainly in the early stages of the interaction. Recently, it has been shown that SLs promote infection thread formation influencing the bacterial partner (McAdam et al., 2017). Nevertheless, the function of the SL transporter in the nodule, like in the root, could be the removal of SLs from the site of biosynthesis to avoid SL accumulation in the meristem and/or deliver them to the place of perception. It cannot be excluded that a redundant transporter can take over the SL transport under LRS. Analysis of publicly available transcriptomic data showed that in Medicago expression of another *PaPDR1* homologue, *MtABCG43*, is induced after Nod factor treatment (van Zeijl et al., 2015b; Jardinaud et al., 2016) and its mRNA was found to accumulate at the highest level in nodules (**Supplementary Figure 7**). It is also possible that in *M. truncatula* SLs do not play an essential role in determining the nodule number. This assumption can be supported by the observation that the silencing of SL biosynthetic gene *MtD27* did not affect the efficiency of nodulation in Medicago (van Zeijl et al., 2015a).

SL acts not only as a signaling molecule in the rhizosphere but also as a phytohormone that adjusts the overall plant architecture to nutrient availability. They control *inter alia* internode elongation (de Saint Germain et al., 2013) and shoot branching by inhibiting axillary bud outgrowth (Gomez-Roldan et al., 2008; Umehara et al., 2008) as well as increasing root surface, and thus, influencing the lateral root and root hair development (Kapulnik et al., 2011; Ruyter-Spira et al., 2011). It is worth noting that *PhPDR1* and *NtPDR6*, in addition to the roots, are expressed in aerial tissues, especially in nodes. Their mutation and/or silencing enhance shoot branching, giving plants a bushy phenotype (Kretzschmar et al., 2012; Xie et al., 2015). Notably, the *PaPDR*-overexpressing Medicago showed several SL-related phenotypes in the shoot, such as deeper leaf margin serrations and increased internode length. Analogous phenotypes were previously reported for Medicago after the exogenous application of *rac*-GR24 at the primary shoot apex. Conversely, SL deficient (*ccd7*, *ccd8*) and insensitive (*d14*) mutants displayed shallower serrations as well as reduced shoot elongation than the WT (Lauressergues et al., 2015). We did not detect expression of *MtABCG59* in aboveground tissues (**Figure 4A**) or observe alterations in the shoot architecture of the *mtabcg59* mutant (**Supplementary Figure 8**). Hence, we propose that in Medicago one or more additional SL transporter(s) can operate to control aerial developmental traits. One of the candidates might be MtABCG44 (71% amino acid identity to MtABCG59) that exhibits a broad expression pattern (**Supplementary Figure 7**).

## Conclusion

In conclusion, in the present study we have identified and characterized MtABCG59, an ABC class G transporter involved in SL export towards the soil that positively affects arbuscular mycorrhiza formation in *Medicago truncatula*. We propose that its primary function is releasing SLs into the rhizosphere to attract AMF during the presymbiotic stage of interactions. The root specific expression of *MtABCG59* and the lack of *mtabcg59* aboveground phenotypes suggests that in *M. truncatula*, SL distribution can be mediated by more than one transporter. Moreover, with the overexpression of the petunia SL transporter PaPDR1 in *M. truncatula*, we have demonstrated the importance of ABCG proteins for the translocation of orobanchol-type molecules, regardless of root anatomy and phylogenetic relationships.

## Data Availability Statement

Sequence data from this article can be found in the GenBank database under the following accession numbers: MTR_3g107870 for MtABCG59, MTR_1g011640 for MtABCG43, MTR_1g011650 for MtABCG44, MTR_3g095530 for Mtactin, AFA43815 for PaPDR1, MTR_1g028600 for MtPT4, JQ292812 for PaPDR1.

## Author Contributions

JB and LB conceived and designed the study. JB, LB, and NS performed the experiments. JB and EM secured funding for the project. JB, MJ, LB, and EM analyzed and interpreted the data. JB wrote the manuscript (initial draft preparation). MJ, LB, and EM wrote the manuscript (critical editing and review of the manuscript).

## Funding

National Science Centre grant number: SONATA 2015/17/D/NZ3/03625. Swiss National Foundation grants number 31003A-152831 and 31003A-169546.

## Conflict of Interest

The authors declare that the research was conducted in the absence of any commercial or financial relationships that could be construed as a potential conflict of interest.

## Abbreviations

AM, arbuscular mycorrhiza; HPC, hypodermal passage cell; LRS, legume-rhizobium symbiosis; SL, strigolactone.
