# Supplementary material for: The Full-Size ABCG Transporter of Medicago truncatula Is Involved in Strigolactone Secretion, Affecting Arbuscular Mycorrhiza
Source: Front Plant Sci. 2020 Feb 7;11:18. doi: 10.3389/fpls.2020.00018 (PMC7019051; doi:10.3389/fpls.2020.00018)
Supplement: Supplementary file 1 [file DataSheet_1.pdf]

Supplementary Material

Supplementary Table 1. List of primers used in this study

A. Primers for real-time PCR analyses

**Genes**	**Primers**
MtABCG59	5'-GCATTTGGACAGGTTGCTG-3'<br>5'-GGTTCCATAGCAAGTAGAAGC-3'
MtABCG43	5'-CCCTTATCTAGAATACCAATATGG-5'<br>5'-TGAAGAGAAGCATTGGAGTG-3'
MtABCG44	5'-GCAGCACACAGGTTATTATAGAG-3'<br>5'-GAATGAGGAGGACAGTATTGG-3'
MtPT4	5'- TCCGCGAAATCTGTTATGATAACCCT-3'<br>5'- AACTGCTGCTATGAAAGCGCCT-3'
*MtBCP1**	5'-CCGGAAAGGACGTTATTCAA-3'<br>5'-TGGCTACCATGATGACTCCA-3'
MtCCD7	5'-TATCTCTATGCTGCAACCAC -3'<br>5'-CTCAACAACAAGAAGGTAACC-3'
MtCCD8	5'-GATTGCCAGAGCTAAGTTCC-3'<br>5'-ATGGATTGCTATCAGCTTGC-3'
Mt-Actin	5'-GTACTTTCCAGCAGATGTGG-3'<br>5'-AACCTACAGACATCCAGTGG-3'

B. Promoter activity analyses (gene specific sequences underlined)

**DNA fragments**	**Primers**
PrABCG59-GUS<br>(*Eco*RI/*Hind*III)	5'-atgaattcaTTCATATTCAAGATAGAGTCG-3'<br>5'-actaagcttGTATGTATTATTTTGTATTATGTACTC-3'
PrABCG59-NLS-GFP	5'-tagttggaatgggttcgaaTTCATATTCAAGATAGAGTCG-3'<br>5'-ttatggagttgggttcgaaGTATGTATTATTTTGTATTATGTACTC-3'

C. Primers used for genotyping

**DNA fragments**	**Primers**
NF12356 WT	5'-GTTCAATGGATACTCCGAGC-3'<br>5'-AACGGCACTAACAAGTTGC-3'
NF12356 *mtabcg59*	5'-GTTCAATGGATACTCCGAGC-3'<br>5'-GCTACCAACCAAACCAAGTC-3'
NF15758 WT	5'-GTCATAGCCTTCTTGTTCGG-3'<br>5'-GGTTCCATAGCAAGTAGAAGC-3'
NF15758 *mtabcg59*	5'-GCTACCAACCAAACCAAGTC-3'<br>5'-GGTTCCATAGCAAGTAGAAGC-3'
PaPDR1	5'-TGGAATGTATTCAGCTATGCC-3'<br>5'-AAGAATGAGAGCAACATATCCC-3'

D. Primers used for cloning of *MtABCG59* gDNA (gene specific sequences underlined)

DNA fragment	Primers
*MtABCG59 SalI/Not*I	5'-gcgtcgacATGGAAGGTGGTGAACTGAG-3'<br>5'-aagcggccgcTACAATTTGGACAA AATGCTTATC-3'

E. Primers flanking the T-DNA insertion sites

DNA fragment	Primers
mtabcg59 mutants	5'-GTTCAATGGATACTCCGAGC-3'<br>5'-GGTTCCATAGCAAGTAGAAGC-3'

* primers from (Zhang et al., 2015)

Supplementary Table 2. Optimized fertilizer solution concentrations for legumes*

Compound	Final concentration
$MgSO_4$	2 mM
K_2SO_4	4.9 mM
KCl	1.3 mM
KH_2PO_4	2 mM
KNO_3	10 mM
$NH_4H_2PO_4$	2 mM
$Ca\ (NO_3)_2$	2 mM
$FeSO_4$	0.1 mM
Na_2EDTA	0.1 mM
$MnSO_4$	83 µM
$MnCl_2$	23 µM
KJ	5 µM
$ZnSO_4$	30 µM
$CoCl_2$	0.1 µM
$CuSO_4$	0.32 µM
H_3BO_3	0.1 µM
Na_2MoO_4	1 µM

* modified PFN nutrient solution (Strozycki et al., 2003).

Supplementary Table 3. Accession numbers of the *Medicago truncatula* full-size *ABCG* genes (*PDRs*)

Name	***Name****	***Locus***
MtABCG37	MtPDR1	medtr7g098750
MtABCG38	MtPDR2	medtr7g098740
MtABCG39	MtPDR3	medtr7g098760
MtABCG40	MtPDR4	medtr7g098300
MtABCG41	MtPDR5	medtr7g098800
MtABCG42	MtPDR6	medtr7g098780
MtABCG43	MtPDR7	medtr1g011640
MtABCG44	MtPDR8	medtr1g011650
MtABCG45	MtPDR9	medtr2g102640
MtABCG46	MtPDR10	medtr2g102670
MtABCG47	MtPDR11	medtr2g102660
MtABCG48	MtPDR12	medtr7g104100
MtABCG49	MtPDR13	medtr7g104110
MtABCG50	MtPDR14	medtr7g104130
MtABCG51	MtPDR15	medtr4g123850
MtABCG52	MtPDR16	medtr8g014360
MtABCG53	MtPDR17	medtr5g070320
MtABCG54	MtPDR18	medtr2g101090
MtABCG55	MtPDR19	medtr4g113070
MtABCG56	MtPDR20	medtr7g098320
MtABCG57	MtPDR21	medtr7g098370
MtABCG58	MtPDR22	medtr7g098890
MtABCG59	MtPDR23	medtr3g107870
MtABCG60	MtPDR24	medtr7g104150

* MtPDR1-MtPDR24 (Banasiak and Jasinski, 2014), Medicago PDR proteins were renamed according to (Verrier et al., 2008).

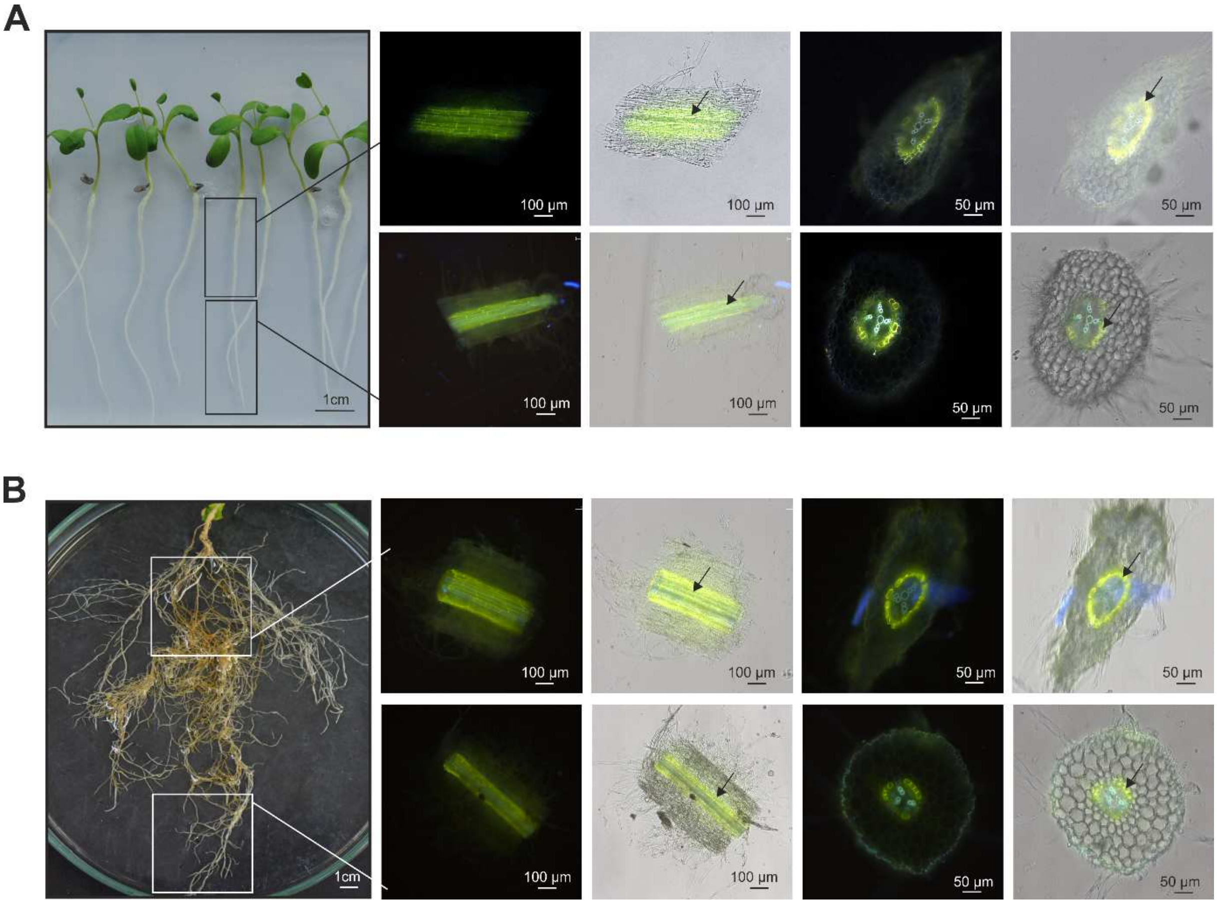

Supplementary Figure 1. Suberin staining in *Medicago truncatula* roots. **(A-B)** Fluorol Yellow 088 staining of suberin only within the endodermis in roots of 7-day-old *Medicago truncatula* seedlings grown on agar plates **(A)** and the roots of 6-week-old *M. truncatula* grown in pots **(B)**. Rectangles indicate the position in the root that the fragments/sections were sourced from.

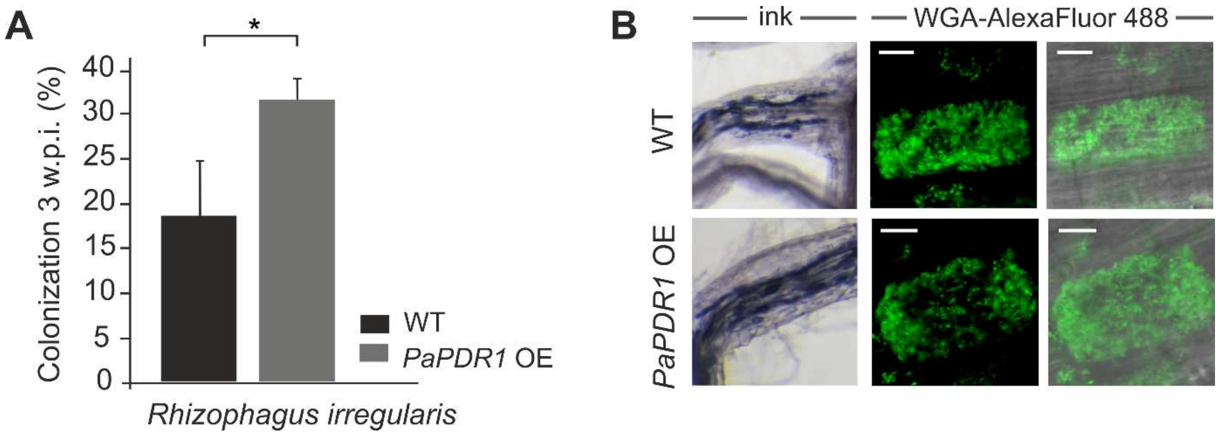

Supplementary Figure 2. Mycorrhizal phenotype of the *Medicago truncatula* plants overexpressing (OE) the *PaPDR1*. (A) Mycorrhizal colonization of *M. truncatula* control (WT) and *PaPDR1* OE roots 3 weeks after inoculation with *Rhizophagus irregularis.* The percentage of the root length colonized by the mycorrhizal fungi is shown. Data represent the means ± SE of three independent biological experiments (i.e. 3 pools of 3 plants each). Significant differences from the control plants were determined by Student's t-test and are indicated by: * $P < 0.05$. **(B)** Arbuscules formed in the WT and *PaPDR1* OE plants were morphologically similar. Ink-staining of fungal structures (left panel), WGA-AlexaFluor 488 staining of arbuscules (right panel), scale bar, 10 µm.

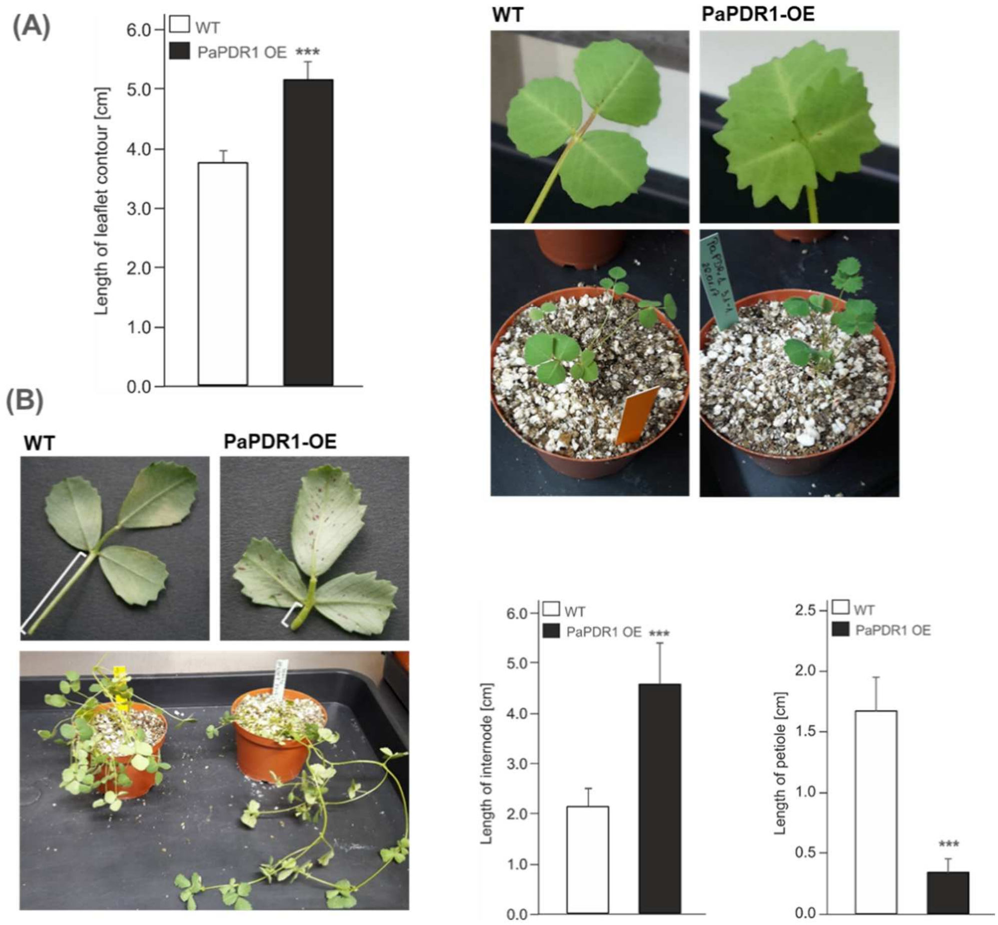

Supplementary Figure 3. Aboveground phenotype of the *Medicago truncatula* plants overexpressing (OE) *PaPDR1*. (A) Leaflet serration phenotypes of the 3-week-old plants. Length of the leaflet contour corresponds to the mean ± SD of 6 leaflets. **(B)** Shoot architecture of two-month-old plants. Length of the internodes and petioles correspond to the mean ± SD of n = 20 and n = 25, respectively. Significant differences from the WT plants were determined by Student's t-test and are indicated by: *** P < 0.001.

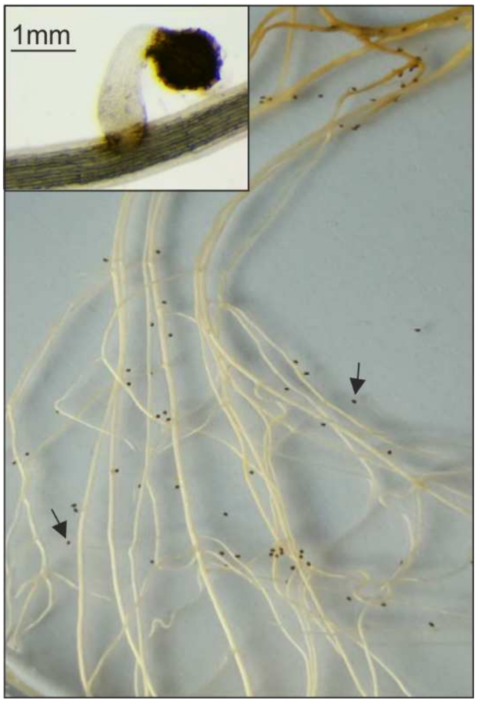

Supplementary Figure 4. Rhizotron Petri dish system. Germination of *Phelipanche ramosa* seeds exposed to Medicago roots exudates. The off root seeds are shown by arrows and the zoom-in picture represents on root germinating seed.

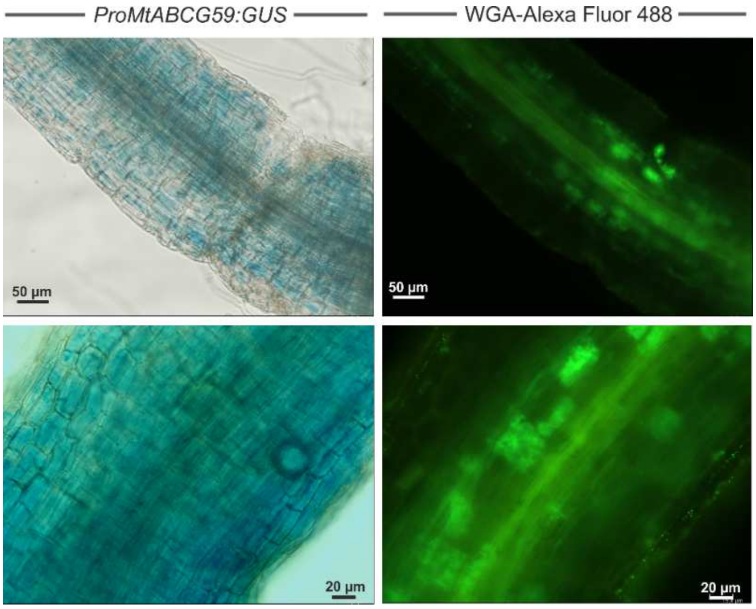

Supplementary Figure 5. Promoter activity analyses of *MtABCG59* in 4-week-old mycorrhized *Medicago truncatula* roots expressing *ProMtABCG59:GUS*. Fungal structures were visualized by staining roots with WGA-AlexaFluor 488.

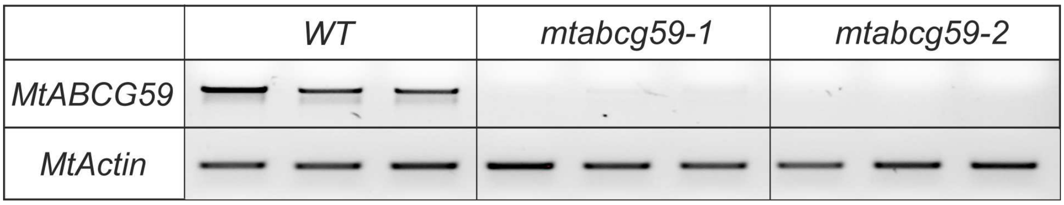

Supplementary Figure 6. Identification of *mtabcg59* mutants. RT-PCR analysis using a pair of primers flanking the T-DNA insertion sites did not detect *MtABCG59* expression in *mtabcg59-1* and *mtabcg59-2*, indicating that both of them are null alleles.

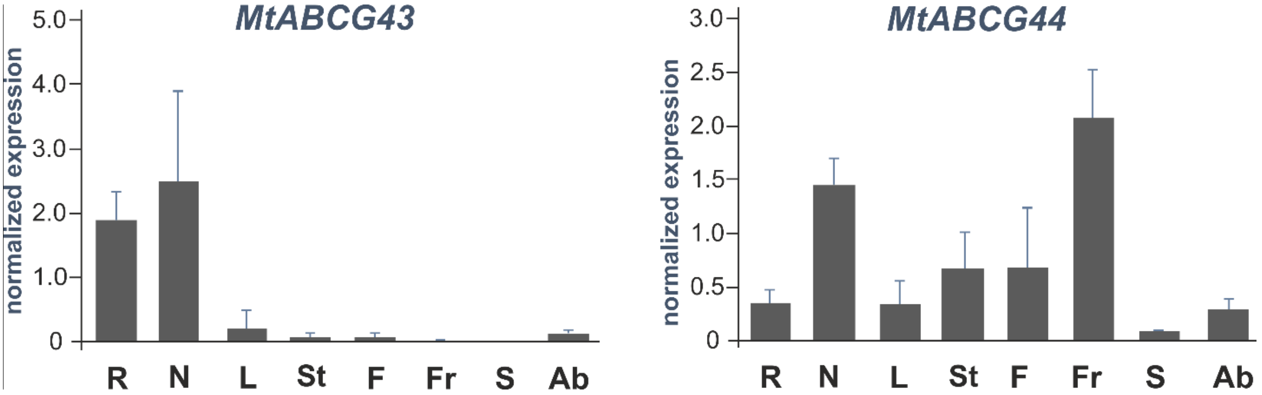

Supplementary Figure 7. Quantitative PCR expression analysis of *MtABCG43* and *MtABCG44* in *Medicago truncatula* organs (R-root; N-nodule; L-leaf; St-stem; F-flower; Fr-fruit; S-seed; Ab-axillary bud). The transcript levels were normalized to the actin gene. Data represent the mean ± SD of three independent biological experiments and three technical repeats.

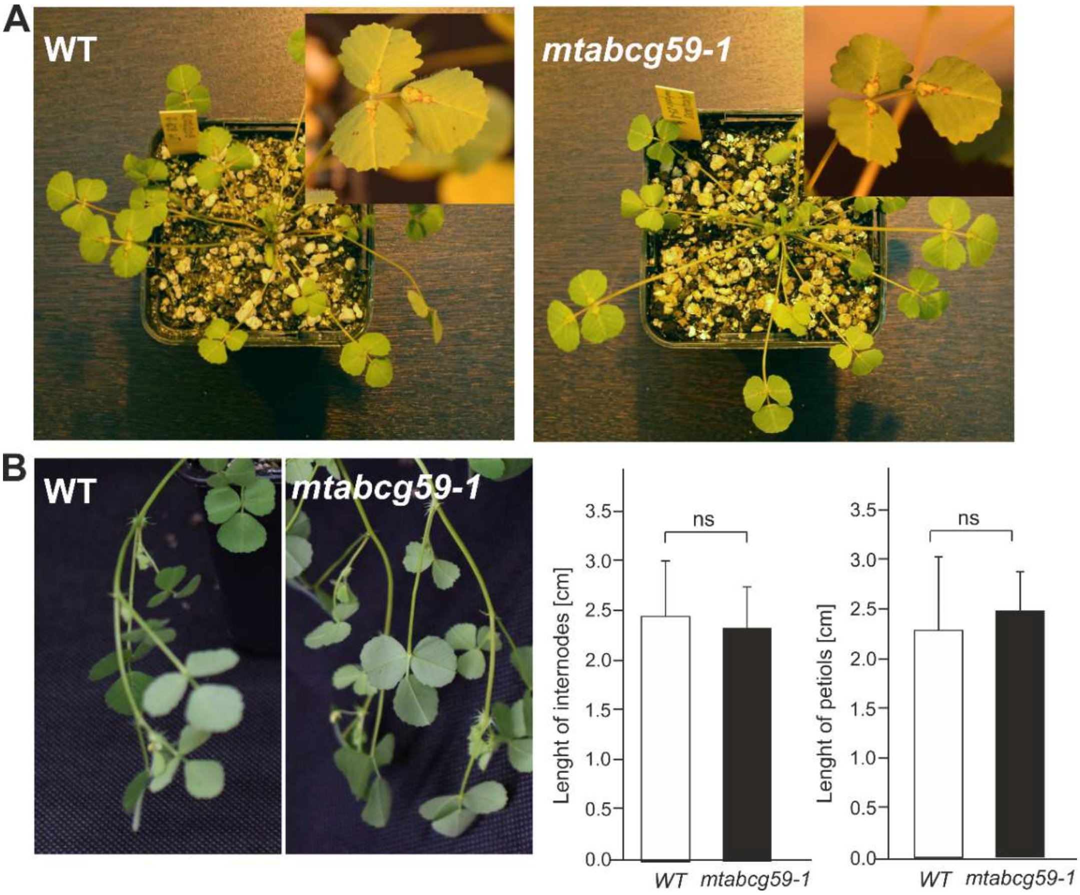

Supplementary Figure 8. Lack of aboveground phenotype of *mtabcg59* plants. (A) No differences in the leaf margin serrations between 4-week-old *WT* and *mtabcg59-1* plants. **(B)** Shoot architecture of the six-week-old plants. No differences in the internode and petiols length between 4-week-old *WT* and *mtabcg59-1* plants. Length of internodes and petioles corresponds to the mean ± SD of n = 20.

Supplementary Reference

Banasiak, J., and Jasinski, M. (2014). Defence, Symbiosis and ABCG Transporters. *Plant Abc Transporters*, 163-184. doi: 10.1007/978-3-319-06511-3_9.

Strozycki, P.M., Skapska, A., Szczesniak, K., Sobieszczuk, E., Briat, J.F., and Legocki, A.B. (2003). Differential expression and evolutionary analysis of the three ferritin genes in the legume plant Lupinus luteus. *Physiologia Plantarum* 118(3), 380-389. doi: DOI 10.1034/j.1399-3054.2003.00081.x.

Verrier, P.J., Bird, D., Burla, B., Dassa, E., Forestier, C., Geisler, M., et al. (2008). Plant ABC proteins--a unified nomenclature and updated inventory. *Trends Plant Sci* 13(4), 151-159. doi: 10.1016/j.tplants.2008.02.001.

Zhang, X., Pumplin, N., Ivanov, S., and Harrison, M.J. (2015). EXO70I Is Required for Development of a Sub-domain of the Periarbuscular Membrane during Arbuscular Mycorrhizal Symbiosis. *Curr Biol* 25(16), 2189-2195. doi: 10.1016/j.cub.2015.06.075.
